# Statin-Associated Immune-Mediated Necrotizing Myopathy With Dual Anti-3-Hydroxy-3-Methylglutaryl-Coenzyme A Reductase (Anti-HMGCR) and Anti-OJ Positivity: A Case Report of Fulminant Weakness and Respiratory Failure

**DOI:** 10.7759/cureus.96123

**Published:** 2025-11-05

**Authors:** Mohammad Ashiqur Rahman, Deena John, Tehreem Khan, Muhammad Farooq, Priyangika Hidelaratchi

**Affiliations:** 1 Respiratory Medicine, University Hospitals of Leicester NHS Trust, Leicester, GBR

**Keywords:** anti-hmgcr myopathy, anti-oj myopathy, immune-mediated necrotizing myopathy, immune mediated necrotizing myopathy (imnm), life support non invasive ventilation, myopathy associated respiratory failure, overlap myopathy, pulmonology, rheumatology, statin associated myopathy.

## Abstract

Immune-mediated necrotizing myopathy (IMNM) is a rare and aggressive form of idiopathic inflammatory myopathy, which is often associated with statin exposure and myositis-specific antibodies such as anti-HMGCR or anti-synthetase antibodies. It is characterized by rapidly progressive muscle weakness, markedly elevated creatine kinase (CK), and often significant morbidity due to respiratory or bulbar involvement.

We share the story of a 71-year-old man with type 2 diabetes mellitus, asthma, chronic kidney disease, and hypercholesterolemia who presented with progressive weight loss and difficulty in walking. He was on Atorvastatin for elevated cholesterol levels. His condition rapidly worsened, leading to lower limb weakness, dysphagia, and recurrent type 2 respiratory failure, requiring prolonged non-invasive ventilation. Electromyography, lower limb MRI, showed features of polymyositis, and biopsy confirmed IMNM. Serology was positive for anti-HMG-CoA (3-hydroxy-3-methylglutaryl-coenzyme A) reductase and anti-OJ antibodies. He was treated with high-dose corticosteroids, mycophenolate, and intravenous immunoglobulin. His hospital course was complicated by aspiration pneumonia, paroxysmal atrial fibrillation, and severe dysphagia requiring gastrostomy feeding. With intensive multidisciplinary care, including respiratory, rheumatology, physiotherapy, and speech therapy, he gradually improved in respiratory function and mobility, regaining upper limb strength and partial independence at discharge.

This case demonstrates the diagnostic complexity of IMNM, the need for early recognition in patients with statin exposure and rapidly progressive weakness, and the importance of aggressive immunosuppression alongside comprehensive multidisciplinary care. Despite multiple complications, meaningful functional recovery is possible with timely and coordinated management.

## Introduction

Immune-mediated necrotizing myopathy (IMNM) is a rare and severe subtype of idiopathic inflammatory myopathies (IIMs), which is characterized by progressive proximal muscle weakness, significantly elevated creatine kinase (CK) levels, and histopathological evidence of myofiber necrosis with minimal inflammatory infiltrates [1]. It is often distinguished from other inflammatory myopathies, such as dermatomyositis and polymyositis, by its more aggressive clinical course and characteristic muscle biopsy findings of muscle fiber necrosis, regeneration, and minimal lymphocytic inflammation [1]. 

IMNM is typically associated with specific autoantibodies, most commonly anti-3-hydroxy-3-methylglutaryl-coenzyme A reductase (HMGCR) and anti-signal recognition particle (SRP) antibodies [1]. Less commonly, anti-synthetase antibodies, such as anti-OJ, may also be detected, either in isolation or in overlap syndromes. Anti-OJ antibodies, directed against Iso leucyl-tRNA synthetase, are usually linked with anti-synthetase syndrome, a condition associated with interstitial lung disease, arthritis, and mechanic’s hands, which is presented as thickened, cracked skin on the hands, resembling the hands of a manual laborer. However, their association with necrotizing myopathy is rare and not well understood [2,3]. 

Clinical manifestations of IMNM can be severe, including profound weakness, dysphagia, and respiratory compromise requiring ventilatory support [1,2]. The diagnosis relies on a combination of clinical presentation, serological testing, imaging, electromyography (EMG), and muscle biopsy [2,4]. Early recognition and initiation of immunosuppressive therapy are essential to improve long-term outcomes [2,5]. 

We present a complex case of anti-HMGCR and anti-OJ antibody-positive IMNM in an elderly patient with a rapidly progressive disease course complicated by recurrent type 2 respiratory failure, severe dysphagia, and prolonged non-invasive ventilatory support. This case highlights the diagnostic challenges and importance of a multidisciplinary approach in managing atypical and aggressive presentations of IMNM [3,5]. 

## Case presentation

A 71-year-old man with a medical history of type 2 diabetes mellitus, asthma, chronic kidney disease, and hypercholesterolemia was referred to the hospital with progressive weight loss and difficulty in walking for over 2 months. He was previously fit and independent in his mobility. He did not have any preceding history of fever, muscle pain, or infectious symptoms. There was no personal or family history of malignancy. There was no tobacco smoking history. His home medications included Insulin, Ramipril, Metformin, Atorvastatin, Montelukast, Budesonide, Formoterol fumarate dihydrate (Duoresp spiromax) inhaler, and Empagliflozin. He had been on Atorvastatin 20 mg daily, started three years ago, without any dose change or break in his statin therapy. Physical examination was unremarkable except for neurological findings of proximal muscle weakness in the lower limbs with reduced muscle mass. 

Initial laboratory investigations revealed elevated inflammatory markers and deranged liver function tests (Table 1). A chest X-ray was unremarkable, and blood and urine cultures were negative.

**Table 1:** Routine blood tests during admission. ALP, alkaline phosphatase; CRP, C-reactive protein; ALT, alanine aminotransferase

Test	Result	Normal range
WBC (×10⁹/L)	24.3	4.0-11.0
Hb (g/L)	116	130-180
Platelet (×10⁹/L)	334	140-400
Neutrophil (×10⁹/L)	23.13	1.50-7.50
CRP (mg/L)	56	<5
ALT (U/L)	183	10-49
Total bilirubin (µmol/L)	14	0-21.0
ALP (U/L)	130	30-130

**Figure 1:**
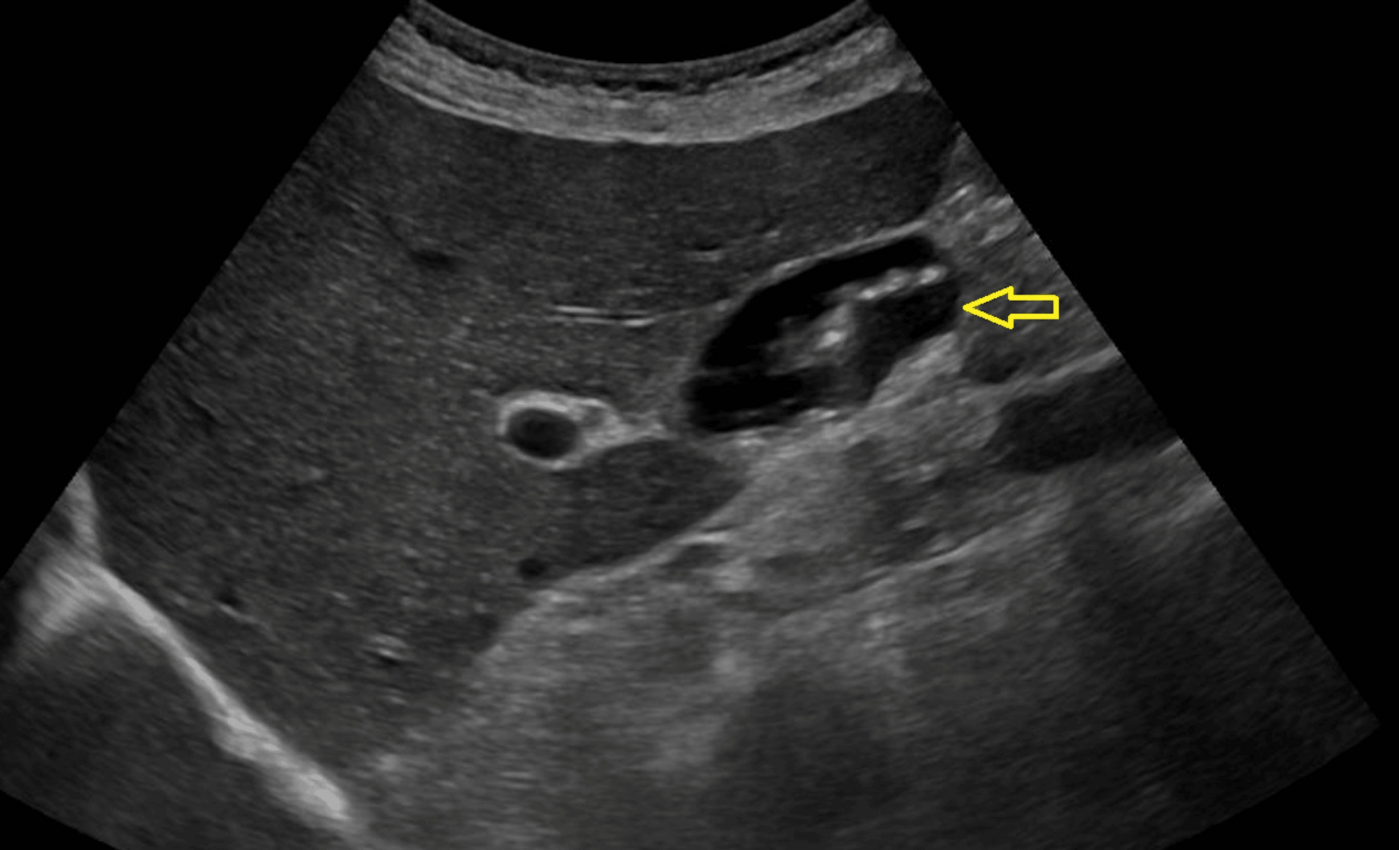
Ultrasound of the abdomen showing gall stone (arrow).

An ultrasound of the abdomen revealed multiple non-obstructive gallstones, and a CT scan of the neck, thorax, abdomen, and pelvis did not reveal any pathology to explain his symptoms (Figure 1). 

Autoimmune profile, liver antibody screen, non-invasive liver screen, and bloodborne virus (BBV) screen were unremarkable. CK level was markedly elevated at 3,858 U/L. Myositis antibody panel, MRI of the lower limbs, and electromyography (EMG) were requested. Atorvastatin was discontinued. 

The patient’s myositis profile is presented in Table 2.

**Table 2:** Myositis antibody panel results.

Antibody (blot)	Result
Anti-Mi2 alpha	Not detected
Anti-Mi2 beta	Not detected
Anti-TIF1 gamma	Not detected
Anti-MDA5	Not detected
Anti-NXP2	Not detected
Anti-SAE1	Not detected
Anti-Ku	Not detected
Anti-PM-Scl100	Not detected
Anti-PM-Scl75	Not detected
Anti-Jo-1	Not detected
Anti-SRP	Not detected
Anti-PL-7	Not detected
Anti-PL-12	Not detected
Anti-EJ	Not detected
Anti-OJ	Detected
Anti-Ro-52	Not detected

**Figure 2:**
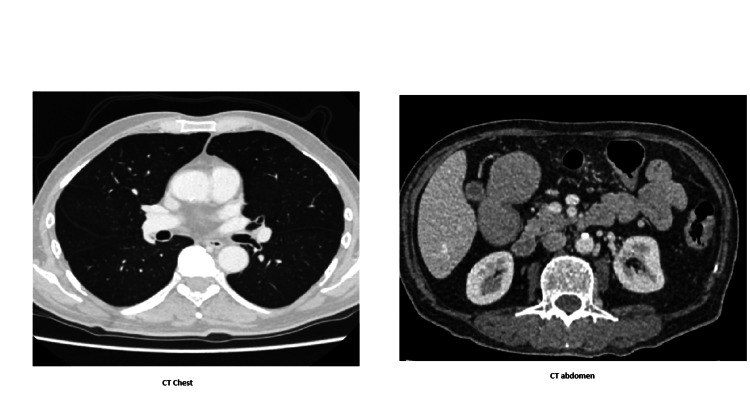
CT of the chest and abdomen showed normal findings.

**Figure 3:**
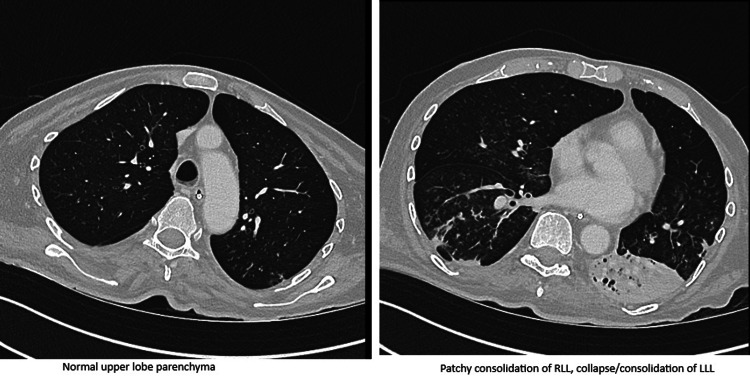
CT scan of the chest shows patchy consolidation in the right lower lobe and collapse/consolidation in the left lower lobe. Otherwise, the lung parenchyma is normal, with no interstitial lung disease (ILD) changes.

Despite initial management, the patient's muscle weakness progressed rapidly, and he became bedbound. He developed a lower respiratory tract infection, likely aspiration-related related and new-onset atrial fibrillation, managed with intravenous antibiotics, anticoagulation, and heart rate control medications. Subsequently, he developed acute type 2 respiratory failure requiring non-invasive ventilation (NIV). Around this time, he also developed severe dysphagia, and nasogastric (NG) tube feeding was initiated. CT imaging ruled out interstitial lung disease and malignancy, but showed bilateral lower lobe infective changes (Figures 2-3).

**Figure 4:**
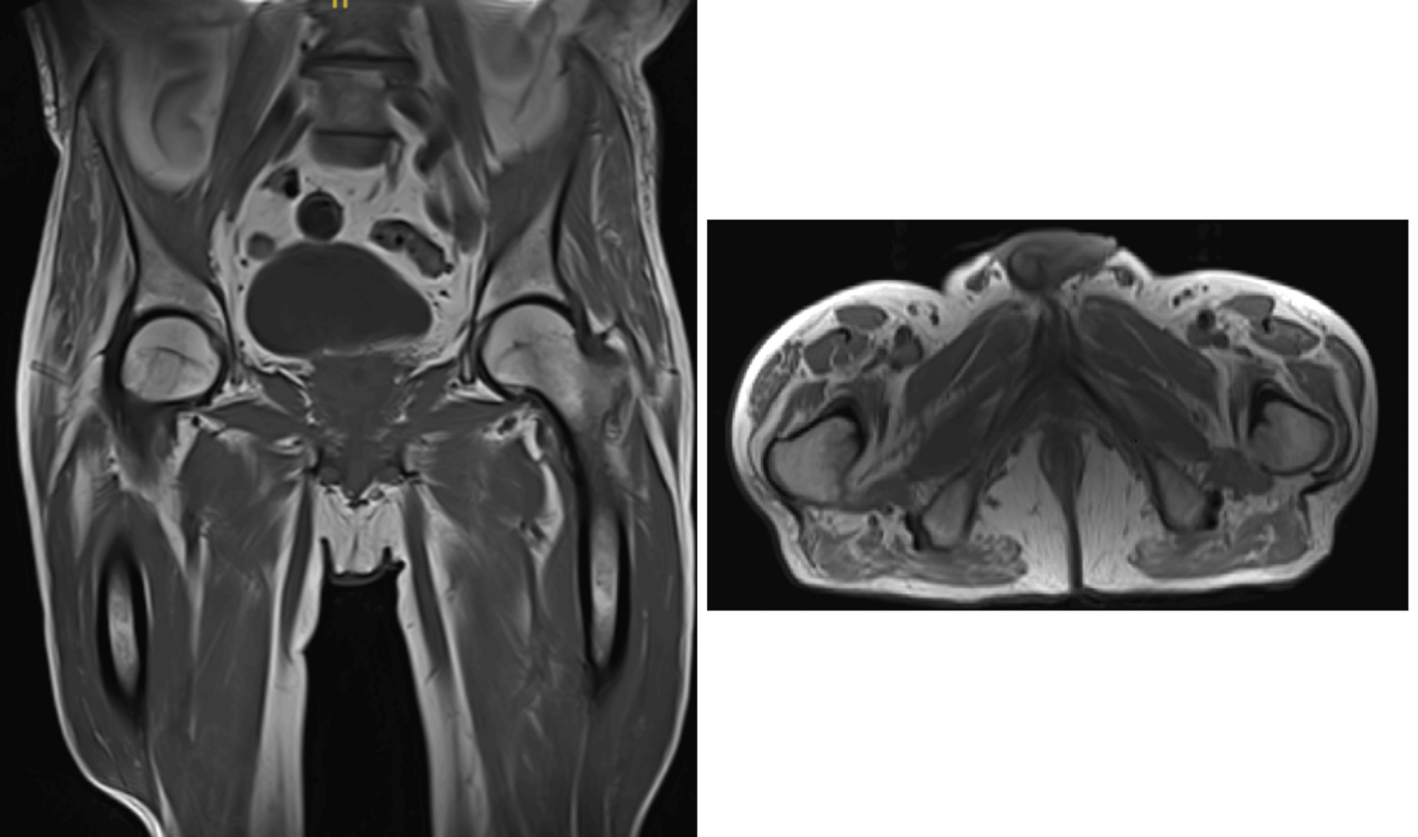
Magnetic resonance imaging (MRI) of the lower limbs showing diffuse polymyositis involving the pelvic and both leg muscles.

**Figure 5:**
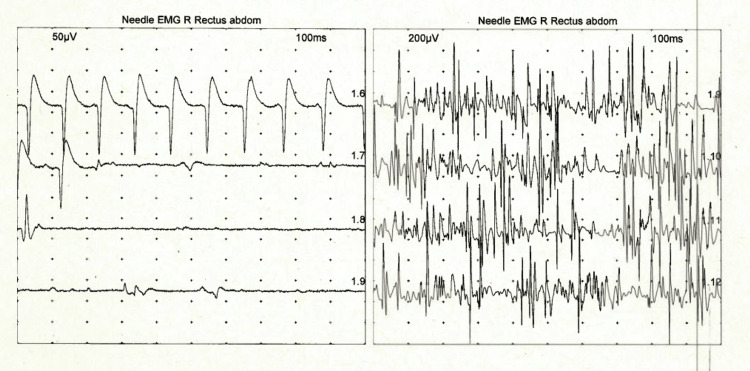
EMG shows small positive sharp waves (PSWs) and polyphasic motor unit potentials with reduced amplitude and duration, confirming polymyositis. EMG, electromyography

MRI of the lower limbs showed inhomogeneous muscle oedema, reported as diffuse polymyositis involving pelvic muscle and both leg muscles (Figure 4). MRI spine was normal. CK remained significantly elevated. EMG showed features of active polymyositis where sensory and motor responses were normal with small, positive sharp waves (PSW), polyphasic, reduced amplitude, and duration, which confirmed diffuse polymyositis (Figure 5). 

The rheumatology team was consulted, and a muscle biopsy was performed from the right quadriceps. He was started on intravenous Methylprednisolone 1gm once daily for three days, followed by oral prednisolone 50 mg for seven days, then tapered 5 mg every seven-day interval. Later, he was added to mycophenolate mofetil.

His condition was further complicated by aspiration pneumonia, which was treated with appropriate antimicrobial therapy. Rheumatologist monitored his clinical progress using the MMT-8 (Manual Muscle Testing) score, which is a standardized method to assess muscle strength in patients with neuromuscular diseases. His initial MMT-8 score was 25/80 on admission.

Muscle biopsy findings were reported as follows: “This skeletal muscle biopsy shows non-specific myopathic features including scattered necrotic fibres, myophagocytosis, regenerating fibres, and complement (C5b9) deposition on endomysial capillaries, but without an inflammatory cell infiltrate or upregulation of MHC class I on non-necrotic fibres. The features are most in keeping with an immune-mediated necrotising myopathy (IMNM).” 

The patient received his first cycle of intravenous immunoglobulin (IVIG). Due to recurrent decompensated type 2 respiratory failure, he required prolonged NIV support and was transferred to a high-dependency unit under respiratory care. Continuous NIV was initiated. 

Further CT imaging ruled out interstitial lung disease and malignancy. The Speech and Language Therapy (SALT) team conducted multiple video swallow assessments, and his feeding was transitioned from NG tube to a radiologically inserted gastrostomy (RIG) tube due to persistent dysphagia. 

Subsequently, serology for HMG-CoA reductase (HMGCR) antibodies also returned positive, confirming a diagnosis of dual anti-HMGCR and anti-OJ antibody-positive IMNM (Table 3). The physiotherapy team was actively involved in his rehabilitation throughout his hospital stay. His blood glucose levels were initially erratic but later stabilized with input from the diabetes team.

**Table 3:** Anti-HMG-CoA reductase test. HMG-CoA, 3-hydroxy-3-methylglutaryl-coenzyme A

Antibody tested	Result
Anti-HMG-CoA reductase (HMGCR)	151 CU/mL (normal range 0-14.9)

**Figure 6:**
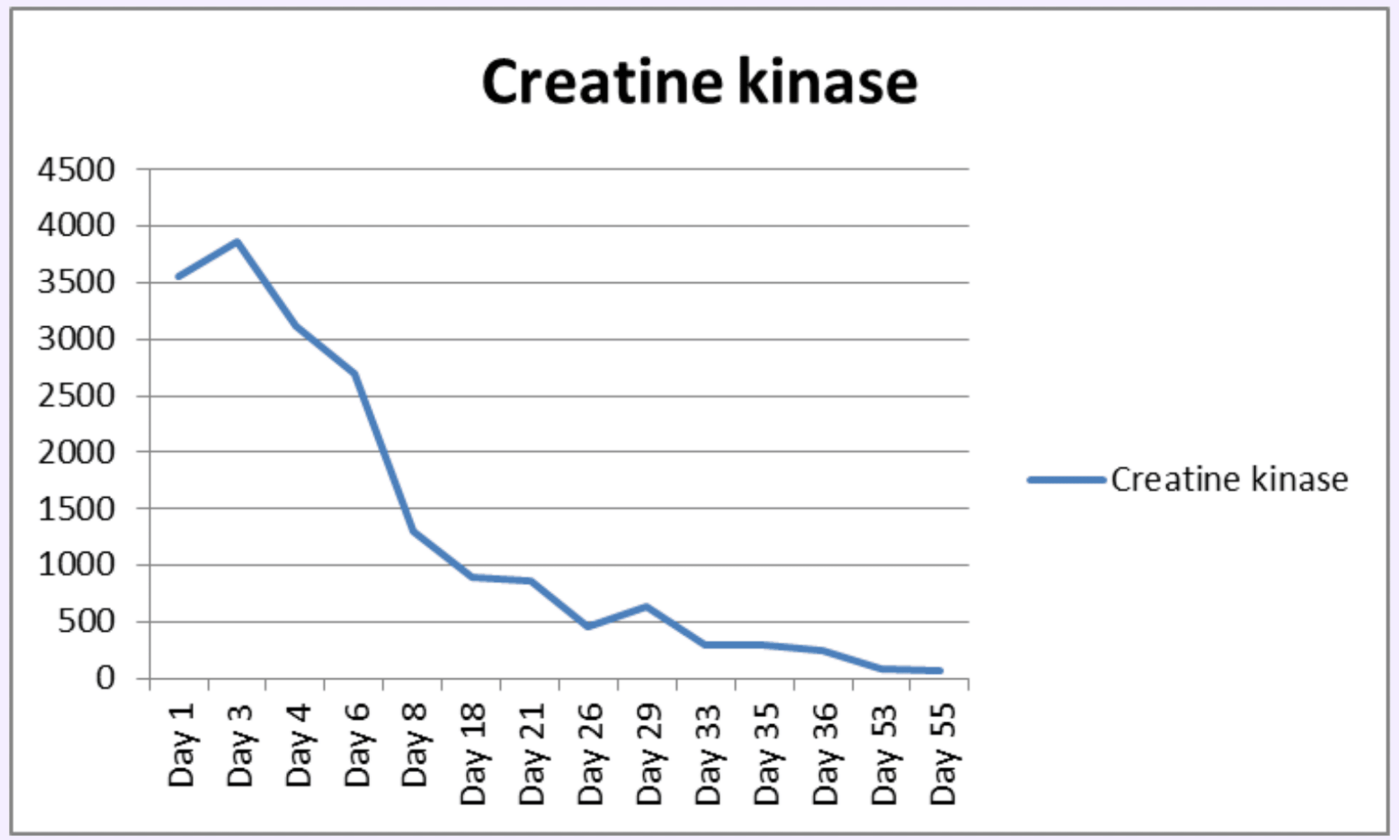
Gradually declining CK levels indicating a response to treatment. CK, creatine kinase

The patient remained on continuous NIV for approximately two months. Gradual clinical improvement was observed, as evidenced by monitoring of the CK level, as evidenced by a downward trend in CK levels, which decreased from a peak of 3,858 to 96 U/L over this period (Figure 6). A second cycle of IVIG was administered following the advice of the rheumatology team as unable to wean form NIV. Gradual weaning from continuous NIV was successfully achieved, and the patient subsequently regained upper limb strength with the ability to self-feed. His Manual Muscle Testing-8 (MMT-8) score improved from 25/80 to 50/80 by the time of discharge.

At the time of discharge, he remained on a domiciliary NIV regimen, used primarily overnight and during the day as required. He continued immunosuppressive therapy with prednisolone and mycophenolate, with close outpatient follow-up by the rheumatology, respiratory, and physiotherapy teams. At the two-month review, he showed marked improvement, including the ability to stand with assistance using a rotunda. His NIV requirements had reduced significantly, limited to overnight use and a few hours during the day. 

## Discussion

Autoimmune myopathies are rare, acquired disorders of muscles that occur due to immune-mediated damage to muscle fibers. They can be classified as inflammatory myopathies, such as polymyositis and dermatomyositis, and immune-mediated necrotizing myopathies. The IMNMs are divided into three subtypes: anti-SRP IMNM, anti-HMGCR IMNM, and seronegative IMNM [6]. The overall prevalence of autoimmune myopathies is 9 to 14 cases per 10,000 [7]. This case presents a diagnostically and therapeutically challenging instance of IMNM in an elderly patient with dual positivity for anti-HMGCR and anti-OJ antibodies, further complicated by progressive respiratory failure and severe dysphagia.

The presence of both anti-HMGCR and anti-OJ antibodies in this patient is unusual and noteworthy. Anti-HMGCR antibodies are typically associated with statin exposure and are the most common antibodies linked to IMNM. Anti-HMGCR immune-mediated necrotizing myopathy usually presents with a subacute onset of progressive proximal muscle weakness with a rise in creatinine kinase levels [6,7]. Although dysphagia and respiratory muscle involvement can be symptoms in some patients with anti-HMGCR IMNM, skin involvement and association of ILD with anti-HMG COA IMNM are extremely rare, unlike their association with other inflammatory myopathies, such as polymyositis and dermatomyositis [6,8,9]. 

Anti-OJ antibodies, on the other hand, are one of the antisynthetase antibodies commonly associated with antisynthetase syndrome (ASS), a condition characterized by interstitial lung disease (ILD), arthritis, Raynaud’s phenomenon, mechanic’s hands, and myositis. Although this patient did not exhibit classic features of ASS or ILD on imaging, the presence of anti-OJ antibodies may have contributed to the disease severity and systemic involvement, including his severe respiratory compromise and prolonged recovery course. To our knowledge, reports of dual positivity with anti-HMGCR and anti-OJ antibodies are exceedingly rare, with limited data on clinical outcomes or optimal treatment approaches.

The pathogenesis of HMGCR myopathy is explained primarily in correlation with environmental triggers such as direct exposure to statins and consumption of statin-containing foods; immunological factors such as the presence of HLA DRB 11:01 allele, and, more rarely, associations with cancer and viral infections [6,7]. Our patient had a history of uninterrupted statin therapy for three years before this presentation. Statin exposure, particularly in the presence of HLA DRB11:01, causes overexpression of HMGCR in muscle tissue, thereby leading to aberrant processing of antigen-presenting cells on HMGCR proteins, which results in production of cryptic epitopes [6,7]. The binding of statins to HMGCR induces conformational changes in the protein, promoting the release of cryptic epitopes. These cryptic epitopes trigger an autoimmune response by producing HMGCR autoantibodies, which cause complement activation targeting the sarcolemma of the myofiber, ultimately resulting in myofiber injury [6,7]. 

The diagnostic investigations in HMGCR IMNM include creatinine kinase levels, autoantibody tests, muscle MRI, and muscle biopsy [6,7,9]. The intramuscular hyperintensity in STIR sequences in muscle MRI is indicative of autoimmune myopathies; however, differentiating inflammatory from autoimmune myopathies can be difficult. EMG may also demonstrate features consistent with myopathy. Autoantibody testing plays an important role in confirming the diagnosis of immune-mediated necrotizing myopathy. A muscle biopsy is often diagnostic, which reveals evidence of myofiber necrosis and regeneration. The mainstay of treatment involves immunosuppressive agents such as corticosteroids, Methotrexate, Mycophenolate mofetil, Rituximab, Azathioprine, Tacrolimus, cyclosporine, cyclophosphamide, and intravenous immunoglobulin [6,7]. Creatinine kinase enzyme levels serve as a marker of disease activity, and the levels typically normalize with appropriate treatment [6]. Most important for the management is the discontinuation of statin therapy, highlighting that anti-HMGCR-associated IMNM often persists and progresses even after withdrawal of the offending agent. Quantification of muscle strength can also be used as a tool to assess the response to treatment [6].

Prognosis in IMNM is variable and depends on many factors, like age, antibody profile, response to therapy, and systemic involvement. Older age and respiratory muscle weakness are associated with poorer outcomes. This case demonstrates that with aggressive and multidisciplinary care, including respiratory support, nutritional management, immunosuppression, and physical rehabilitation, substantial functional recovery is possible even in severe cases.

## Conclusions

This case highlights the diagnostic complexity and severe clinical course associated with IMNM, particularly in the setting of dual anti-HMGCR and anti-OJ antibody positivity with a previous history of statin exposure. While IMNM typically presents with painless proximal muscle weakness, this patient experienced a rapidly progressive disease complicated by recurrent respiratory failure, severe dysphagia, and prolonged dependence on non-invasive ventilation. The presence of anti-synthetase antibodies, such as anti-OJ, may indicate an overlapping autoimmune process and further complicate the clinical picture. 

Early recognition, prompt immunosuppressive therapy, and comprehensive multidisciplinary management, including respiratory, rheumatology, physiotherapy, and nutritional support, were essential in achieving functional recovery and preventing further complications. This case underscores the importance of considering IMNM in patients with unexplained muscle weakness and elevated CK, even when initial presentations are nonspecific or mimic other systemic conditions.

## References

[1] Allenbach Y, Mammen AL, Benveniste O (2018). Diagnosis and management of immune-mediated necrotizing myopathy. Lancet Neurol.

[2] Milone M (2017). Diagnosis and management of immune-mediated myopathies. Mayo Clin Proc.

[3] Ghannam M, Manousakis G (2020). Case report: Immune mediated necrotizing myopathy with IgG antibodies to 3-hydroxy-3-methylglutaryl-coenzyme A reductase (HMGCR) may present with acute systolic heart failure. Front Neurol.

[4] Benucci M, Terenzi R, Li Gobbi F (2025). Anti-HMGCR-antibody-positive statin-induced myositis: a pilot case series on treatment with bempedoic acid and immunosuppressive therapy. Antibodies (Basel).

[5] Yang M, Yuan J, Wang Y (2024). Treatment of refractory immune-mediated necrotizing myopathy with efgartigimod. Front Immunol.

[6] Pinal-Fernandez I, Casal-Dominguez M, Mammen AL (2018). Immune-mediated necrotizing myopathy. Curr Rheumatol Rep.

[7] Mohassel P, Mammen AL (2018). Anti-HMGCR myopathy. J Neuromuscul Dis.

[8] B.G. Nudelman, J. Valencia Uribe, K. Bhattar, and S. Krishnaswamy (2025). Acute respiratory failure secondary to statin-induced anti-HMG CoA reductase myopathy: a rare complication with diagnostic challenges. Am J Respir Crit Care Med.

[9] Ngo LQ, Wu AG, Nguyen MA, McPherson LE, Gertner E (2016). A case report of autoimmune necrotizing myositis presenting as dysphagia and neck swelling. BMC Ear Nose Throat Disord.

